# Predictability of a New Orthodontic Extrusion Technique for Implant Site Development: A Retrospective Consecutive Case-Series Study

**DOI:** 10.1155/2020/4576748

**Published:** 2020-01-25

**Authors:** E. Conserva, M. Fadda, V. Ferrari, U. Consolo

**Affiliations:** ^1^Department of Surgery, Medicine, Dentistry and Morphological Sciences with Interest in Transplant, Oncology and Regenerative Medicine, University of Modena and Reggio Emilia, Modena, Italy; ^2^Private Practice, Fiesso Umbertiano, Rovigo, Bologna, Italy

## Abstract

In clinical daily practice, there are situations in which implant sites have vertical and/or horizontal bone defects and often we must improve their morphology and dimensions before fixture insertion. It is crucial to carefully evaluate the surgical site as regards the characteristics of both hard and soft tissues. The orthodontic extrusion technique can be used for nonsurgical augmentation of the implant site as an alternative to traditional regenerative/reparative surgical therapies. The orthodontic extrusion is based on a biological mechanism that uses the portion of periodontal ligament, still present on the root before the tooth extraction, for the increase of hard and soft tissues. In the literature, there is no evidence of common guidelines for this technique but only tips based on personal experience and/or previous studies. The aim of this study was to investigate and to validate the reliability of a new orthodontic extrusion technique (MF Extrusion Technique, by Dr. Mauro Fadda) by means of a retrospective consecutive case-series study. After we have done a review of the literature, we evaluated the X-rays of twelve consecutively treated patients before the orthodontic extrusion (T0) and after the stabilization period (T1), in order to quantify, by two different measurements, area and linear, the bone gain obtained by the application of the new technique. All the patients examined showed a significant increase in bone areas with an average value of 31.575 mm^2^. The linear bone gain had an average value of 4.63 mm. Data collected were statistically analysed by the Wilcoxon signed-rank test. The results obtained both from area and linear measurements at T0 and at T1 times showed that there was a statistically significant bone gain with *p* < 0.01.

## 1. Introduction

Bone defects can be treated by different surgical procedures, such as GBR and bone grafts depending on the characteristics of the defect itself. Several techniques are available today by using resorbable and nonresorbable devices. Poli et al. [1] performed a retrospective clinical study in which they assessed the validity of the GBR technique by means of a Ti-mesh filled with intraoral autogenous bone mixed with deproteinized inorganic bovine bone. Herford et al. [2] suggested the use of porcine collagen matrix with the addition of platelet-derived growth factor (PDGF) to accelerate soft tissue healing and promote bone formation. These are invasive techniques with a high degree of morbidity and a risk of failure. Often patients ask for noninvasive procedures. The orthodontic extrusion (OE) can be a valuable nonsurgical technique to obtain a good augmentation of the bone. This technique is based on the stimulation of the biological mechanism of interaction between the fibers of the periodontal ligament (PDL) and the alveolar bone: the complex anchoring system of the periodontal ligament allows the distribution and the absorption, through the alveolar process, of the forces developed during mastication and makes physiological dental movements possible. The alveolar bone is in constant transformation, as elsewhere in the body, being constantly resorbed and rebuilt [3]. The orthodontic movement influences the periodontal anatomical structures and, particularly, the PDL surrounding the root of a natural tooth, which is the key factor inducing the deposition of new bone during the extrusion processes [4]. The extrusive forces create a tension of the PDL fibers, which are strictly connected to the bone, stretching them. This elongation stimulates the osteoblasts to deposit new bone at the alveolar level [3]. The new bone formation occurs on the crestal surface of the alveolar bone and near the root. The periodontal ligament can then be manipulated, producing a predictable biological response, using the controlled movement of the tooth [5, 6].

The technique of orthodontic extrusion for the correction of infrabony defects was described by Brown [7] in 1972. The study showed that only management of soft tissues was not enough to correct the periodontal pockets. The clinician therefore has the opportunity to choose between two alternatives: (1) to surgically eliminate part of the bone crest, through osteoplasty and osteotomy procedures, to reduce or eliminate the infrabony defect; (2) to induce the growth of the periodontal ligament (PDL) that will fill the defect. As first, Brown identified in the extrusion orthodontic movement the technique that can be used to reduce the depth of the pocket, to increase the attachment level of periodontal fibers and to make a change in the bone and in the soft tissue architecture [7]. In 1974, Ingber [8, 9] used the orthodontic extrusion for the so-called “nonrestorable or hopeless teeth” damaged by caries, fractures, or traumas, to restore the ferrule effect. He concluded that orthodontic extrusion is an alternative technique for the management of fractures at the level of the alveolar crest because, thanks to the extrusive forces, there is a stretching of the periodontal fibers with consequent coronal displacement of soft and hard tissues. In fact, Ingber noticed that while the teeth were being extruded, both the bone and the gingiva were more coronally displaced [8, 9]. In 1993, H. Salama and M. Salama [10], inspired by the works of Ingber [8, 9] and Brown [7], published several studies in which orthodontic extrusion was used, no longer to correct a defect of a natural tooth, but to increase hard and/or soft tissues with the aim of improving the morphologic and biologic characteristics of the implant site, before implant insertion. The main concept of their works can be summarized in the sentence “hopeless tooth is not a useless tooth.” In the following years, several authors [11–14] published, especially case reports, with different protocols and results.

The indications for orthodontic extrusion are limited to moderate bone defects characterized by a limited bone resorption that reaches maximum up to the middle third or fourth of the root. In cases where there is a severe circumferential bone loss, orthodontic extrusion cannot create an adequate vertical bone dimension for the ideal positioning of the implant. Even in cases of severe gingival recession associated with bone loss, the final position of the gingival line after extrusion may not be satisfactory for the final aesthetics of the restoration. Finally, orthodontic extrusion is not able to regenerate horizontal bone defects.

Although the literature does not provide a clear information about when to apply orthodontic extrusion techniques, the selected cases generally include teeth that must be extracted for severe bone loss, endodontic lesions, failed apicectomy, or severe root decay. The general contraindications for orthodontic extrusion are presence of chronic and uncontrollable inflammatory lesions, including endoperiodontal combined lesions, fractured roots, inability to control inflammation, and acute infection; the presence of a root hypercemented or in anchylosis because an attempt to extrude an anchylosed tooth would result in an intrusion or unwanted movement of the anchor teeth.

From the literature review, there is no evidence of common guidelines for the orthodontic extrusion but only several tips based on the author's personal experiences [15], and in addition, the method used for measurements is almost never described. The aim of this work was to evaluate and to validate a new orthodontic extrusion technique (MF Extrusion Technique, proposed by Dr. Mauro Fadda), based on a strict clinical protocol, through a retrospective study of twelve consecutive case-series.

## 2. Materials and Methods

### 2.1. Review of the Literature

A systematic search was conducted in PubMed using the following keywords: Orthodontic, Extrusion, Implant Site Development, Forced Eruption, Fibrotomy. The initial research string was [(tooth extrusion OR tooth extraction) OR (forced eruption) OR (orthodontic OR fibrotomy) AND (implant AND site AND development)]. A number of 99 articles were identified. The exclusion criteria were letters, editorials, thesis, articles focusing on orthodontic extrusion for any purpose other than implant site development, studies on the increase of the alveolar bone by means of an orthodontic extrusion without the subsequent insertion of the implant, articles that do not describe the techniques used for orthodontic extrusion, articles that do not describe changes in the hard and/or soft tissue of sites receiving the implant after orthodontic extrusion; and articles not available in English. At the end of this first selection phase, we found 29 full articles for the reading. After they have been carefully read, we excluded two articles which concerned the use of removable equipment; four articles that were not available in full text (we tried to contact authors directly and through Research Gate with no answers); and six articles which concerned aspects of general orthodontics without precise reference to the development of the implant site. At the end, 13 articles were selected (Table 1) well concerning the orthodontic extrusion technique related to the development of the implant site.

### 2.2. Protocol of the New Orthodontic Extrusion Technique

The second part of this work was based on the retrospective consecutive case-series study in which the new technique was used. This extrusion technique, also called MF extrusion technique, was proposed by Fadda and Cortesi [11] in 2009. Patients included had dental elements periodontally or endodontically compromised with serious bone defects and a negative prognosis.

**Figure 1:**
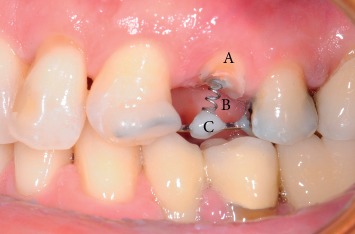
Clinical case #8: tooth 2.4, 150 g force spring cemented into the canal root by resin cement in order to exert the extrusion force along the same tooth axis. Time: T0; A: tooth 2.4 endodontically treated and trimmed; B: 150 g force spring cemented into the canal root; C: square section of an orthodontic steel wire of 0.019 × 0.025 inch anchored to the near teeth and to the spring by composite resin.

**Figure 2:**
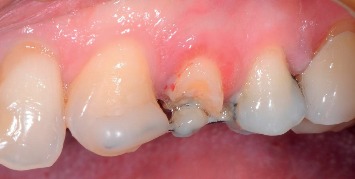
Clinical case #8: tooth 2.4, four-week control. The spring must be activated again, and the tooth needs a selective grinding.

**Figure 3:**
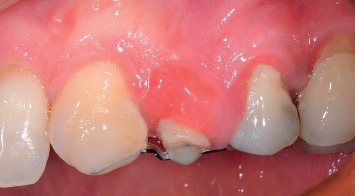
Clinical case #8: tooth 2.4, overcorrection obtained after 12 weeks of stabilization. Time: T1.

They gave a written informed consent to publish their case descriptions and pictures. Before using this new protocol, we used loops or sectional Ni-Ti arches with facial brackets, but we always observed a noncontrolled extrusion direction and especially vestibular dehiscences. If you look at the literature, all the case reports described used these methods. The novelty of the proposed protocol is the use of a 150 g spring that exerts a controlled and controllable force along and concentric to the tooth axis because the spring is directly cemented into the root channel or in the pulp chamber exerting its force through the centre of resistance of the tooth. In consequence of this, we can obtain a bodily movement of the tooth itself, which therefore will not rotate, avoiding the onset of vestibular bone dehiscences, especially in the anterior region. The new extrusion technique protocol used in the study is described as follows:

- Slow orthodontic extrusion speed equal to 1.5/2 mm every two months (0.7–1 mm/month) by using a nickel-titanium spring with a height of 8.00 mm and a tensile force of 150 g cemented into the canal root and anchored to a square section of an orthodontic steel wire of 0.019 × 0.025 inch, by composite resin, metal, or elastic ligature, was fixed with the composite to the buccal or occlusal surface of the adjacent teeth in order to prevent any possible unwanted movement. The steel wire must pass over the resistance centre of the tooth to be extruded in order to well anchor the Ni-Ti spring. To do this, the wire must be properly curved and adapted with orthodontic pliers. The direction of the force must be the same as that of the long axis of the root and must be exerted centred on the root core (Figure 1). The number of elements involved in the anchorage depends on the tooth to be extruded. To ensure an adequate anchoring, the steel wire must be blocked on at least two teeth although at least three teeth or one implant should be involved in the extrusion of the molars.
- Controls were made every four weeks, doing a selective grinding of the tooth crown, only if necessary, so that it does not have any occlusal interference with the antagonist (Figure 2). In the most part of clinical situations, the tooth structure was removed before extrusion started. The need to remove and reactivate the spring depends on the space between the extruding tooth and the device on which the spring is fixed and on the amount of extrusion required. Each time the extruding tooth comes in contact with the device supporting the spring, it must be grinded, the device repositioned and the spring repositioned and reactivated, but, since the teeth to be extruded are usually damaged and hopeless, they can be reduced up to the gingival margin, after pulpectomy, before starting the extrusion, obtaining a space between 4 and 7 mm. If more extrusion is needed (8–14 mm), the spring must be repositioned and reactivated at least once.
- Stabilization of the element for a period of 12 weeks by means of a 0.019 × 0.025 inch square steel orthodontic wire to allow the mineralization of new trabecular bone (Figure 3).
- Before the extrusion, all the patients underwent scaling and root planning and were instructed on the correct home hygiene in order to have a plaque control and minimize the risk of inflammation. In the extrusive phase, maintaining a good periodontal health is essential. For this purpose, we used the airflow technique (Combi-Touch, Mectron SPA, Carasco, Genoa, Italy) with Glycina powder.

**Figure 4:**
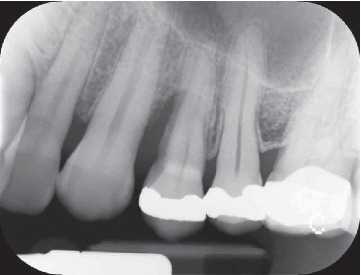
Clinical case #8: male, 42 years old, nonsmoker. X-ray of tooth 2.4 severe bone defect. Time: T0.

**Figure 5:**
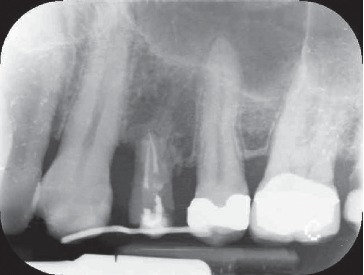
Clinical case #8: tooth 2.4, X-ray after 12 weeks of stabilization. Time: T1.

During the study, two centred intraoral X-rays were taken from all twelve patients: one at time T0 and the second one at time T1 (at the end of the stabilization period) (Figures 4 and 5).

**Figure 6:**
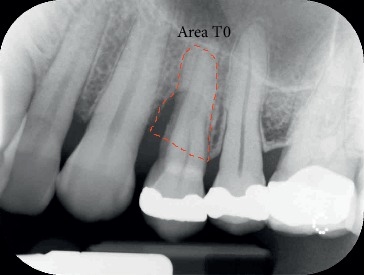
Tooth 2.4: X-ray at T0: the area selected (red contour) represents the part of the root inside both the bone and the defect.

**Figure 7:**
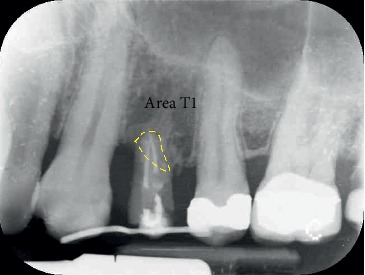
Tooth 2.4: X-ray at T1: the area selected (yellow contour) represents the part of the root inside the bone after 12 weeks of stabilization. The difference between area T0 and area T1 gives the value of bone gain in mm^2^.

To make the X-ray perfectly repeatable and with the least distortion possible, we used a customized resin bite that fitted the patient teeth and that we used for all X-ray controls. The areas selected for the measurements at time T0 and time T1 corresponded to the root portion inside both the bone and the defect: the difference between the area obtained at time T0 and the area obtained at time T1 represented the bidimensional portion of bone augmentation (Figures 6 and 7).

**Figure 8:**
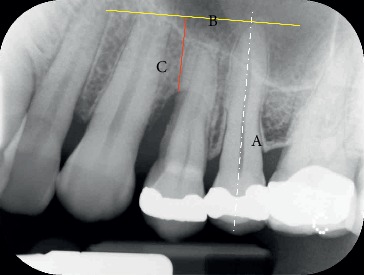
Tooth 2.4: X-ray at T0: the most apical point of the bone defect is marked and its distance (C) from the straight line (B) perpendicular to the long axis of the near tooth (A) and passing through the apex is measured.

**Figure 9:**
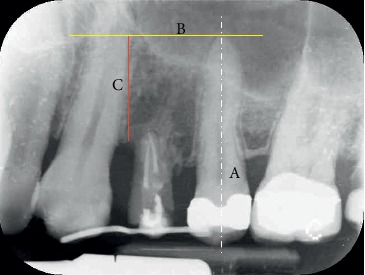
Tooth 2.4: X-ray at T1: the most apical point of the bone defect is marked and its distance (C) from the straight line (B) perpendicular to the long axis of the near tooth (A) and passing through the apex is measured. The difference between the linear measurement T1 and T0 gives the vertical bone gain in mm.

We also measured the linear bone gain in order to compare it with the few data present in the literature. On X-rays at the time T0 and at the time T1, of all twelve cynical cases, the most apical point of the bone defect was marked and its distance from the straight line perpendicular to the long axis of the near tooth and passing through its apex was measured (Figures 8 and 9).

All the measurements were performed using the software Image J (US National Institutes of Health, Bethesda, MD, USA); first of all, the image calibration is performed knowing the dimensions of the plate used for centred intraoral X-rays (30 × 40 mm). We want to underline that the obtained bone gain does not represent only the portion of new bone that has been formed to fill the bone defect but also that portion of bone that has replaced the extruded root portion. All data collected from both kinds of measurements (area and linear) were statistically analysed using the nonparametric Wilcoxon signed-rank test. The significance threshold of the test was set for *p* < 0.01.

## 3. Results

### 3.1. Review of the Literature

Tables 2–4 summarize the results from the analysis of the literature in which different parameters of the different protocols presented in the selected 13 articles were compared, such as extrusion force, extrusion speed, stabilization period, overcorrection, possibly associated fibrotomy, and follow-up recall.

### 3.2. Results of the Retrospective Consecutive Case-Series Study

Table 5 summarizes the results obtained from the measurement of the area of each patient examined before the start of treatment (T0) and at the end of the stabilization period (T1), the mean values, SD, and the gain (ΔT − T1).  Table 6 summarizes the values obtained from the linear measurements of the distance between the most apical point of the bone defect and the traced reference line on the X-rays both at the beginning of the orthodontic extrusion (T0) and at the end of the stabilization period (T1) for each patient examined (Figures 8 and 9). The linear bone gain following the extrusion protocol used has an average value of 4.63 mm, from the minimum of 2.1 mm to the maximum of 7.8 mm. To evaluate whether the bone augmentation obtained by the new orthodontic extrusion protocol was statistically significant, the nonparametric Wilcoxon test of the ranks with sign was used and the significance of the test was set for *p* < 0.01. The results from the statistical analysis of both the area and linear data are shown as follows:

The value of *Z* is −3.0594; the *p* value is 0.00222. The result is significant at *p* < 0.01.

## 4. Discussion

In implant surgery, the alveolus preservation, also called socket preservation, is essential to maintain an adequate bone volume for implant placement and stabilization [26]. Today, various socket preservation techniques are available, using different grafting materials, with or without the use of membranes, to help the clinician to achieve optimal results especially when aesthetic zones are involved. The orthodontic extrusion technique represents a treatment option alternative to the surgery for the socket preservation and for the increase of hard and soft tissues before the implant placement. It is atraumatic and its success depends on the amount of periodontal ligament still present on the tooth root, at least one-third to one-fourth [10, 27]. In addition to the biological and functional advantages, this technique offers a clinical advantage in facilitating the implant placement because the tooth is moved coronally by several millimetres and this movement makes available a greater amount of bone in the apical area of the alveolus after the extrusion process. The residual socket has a very small diameter so that it is easier to prepare the site (no bur bending) especially in cases where the roots are very close to important anatomical structures such as the maxillary sinus or the mandibular nerve. The obtained primary stability, improved as compared to a surgical postextraction site, allows immediate loading of the implant or at least positioning of the healing screw, thus excluding the need for a second surgical phase. From the analysis of the literature, orthodontic extrusion is a technique not widely used, especially for the implant site development, and it showed a variety of different clinical protocols. Somar et al. [28] published a systematic review in which among 491 found articles, only 38 were classified as potentially appropriate and only six were included in the study. This confirms that there is no uniformity of techniques, of protocols and, above all, of bone gain ranging between 3.6 mm and 8 mm. In Table 2, in which the force parameter is evaluated, the forces used during extrusion are ranging from 15 g to >80 g, where the lowest values are normally used for the front elements or monoradicular and highest values for posterior elements.  Table 3 shows that the *extrusion speed* (mm/month) is between 0 and 2 mm per month and, in any case, not more than 2 mm. Higher speeds, obtained using high and rapid forces, are indicated when a coronal tooth movement is desired without a simultaneous movement of the periodontal ligament and therefore without a consequent increase in hard and soft tissue [16, 29]. In our protocol, the speed used during extrusion is less than 1 mm/month, because we must allow the PL cells to organize themselves. The stabilization time (Table 4), following the treatment, is fundamental to allow the new bone trabeculae to mineralize. Most of the studies included in this review indicate the stabilization time in a period ranging between 6 and 24 weeks of retention. In some studies, the stabilization period is based on the mm of extrusion obtained, recommending a period of 1 month for each millimeter extruded [16, 18]. Overcorrection (more extrusion than needed) is recommended to compensate the possible loss of bone and gingiva that may occur as a result of the implant surgery. Brindis and Block [6] (Table 3) suggested a value of 2-3 mm of overcorrection. The technique of fibrotomy (Table 3) is not recommended in any study analysed. The study of Kozlovsky et al. [30] demonstrated that fibrotomy avoids coronal displacement of the gingiva and ligament during tooth extrusion, and Carvalho et al. [31] highlighted how orthodontic extrusion combined with fibrotomy and root planning is indicated when we want to have a crown elongation without alterations in the position of the gingival margin while it is not indicated when you want to coronally move the gingiva and the periodontal ligament together with the tooth. Regarding the follow-up time periods (Table 3), not all studies showed data about it. Longer follow-up time periods ranging from a minimum of one year to a maximum of five years are found only in the studies of Watanabe et al. [22] and Hochman et al. [5]. Amato et al. [19] reports a range of “bone gain” between 0.6 mm and 8 mm on a total of 32 elements analysed while in another study [25] Kwon et al. found that the average bone increase at the interproximal level was 1.36 mm considering 11 elements. Maeda and Sasaki [15] in a case report obtained a bone gain of 4 mm. With our technique, an average linear bone gain of 4.63 mm was obtained, ranging between 2.1 mm and 7.8 mm. This means that the new orthodontic extrusion allowed a linear bone gain equal to or, in most cases, greater than, those obtained with the protocols presented in the literature.

## 5. Conclusions

The orthodontic extrusion for implant site development is a method that can be used successfully as an alternative to surgical techniques. It is very important to have an excellent patient compliance because the time for the extrusion is long (1 mm/month plus three months of stabilization) and to have a very careful oral hygiene in order to avoid any inflammation to obtain a predictable result. In cases of circumferential bone loss, as well as in cases of severe gingival recession, the orthodontic extrusion cannot ensure a vertical bone development suitable for the ideal implant placement, with complications such as bone dehiscence. From the various studies analysed [17, 20, 21, 23, 24], we can conclude that, even if the validity of the orthodontic extrusion technique has been demonstrated, there are no common guidelines that can be followed by the clinicians. On the contrary, there is agreement for the use of mild and continuous forces as well as for the importance of the stabilization period and of the overcorrection. We also found an agreement between the authors for no use of fibrotomy because there is a risk to lose the soft tissue gained. The results obtained with the use of this new orthodontic extrusion protocol, both from area and linear measurements, showed a statistically significant bone augmentation ranging between 2.1 mm and 7.8 mm (4.63 mm mean value) with *p* < 0.01. This means, with the limitations of this two-dimensional analysis based on surface and linear measurements which is not sufficiently accurate such as a 3-D CBCT study, that the new proposed technique could be used by clinicians as a common protocol to perform the orthodontic extrusion technique.

## Figures and Tables

**Table 1:** List of the articles selected after the analysis of the literature and their reference numbers in this article.

Articles selected	Years
H. Salama and M. Salama [10]	1993
Korayem et al. [16]	2008
Brindis and Block [6]	2009
Uribe et al. [17]	2010
Kim et al. [18]	2011
Amato et al. [19]	2012
Rokn et al. [20]	2012
Chou et al. [21]	2013
Watanabe et al. [22]	2013
Hochman et al. [5]	2014
Keceli et al. [23]	2014
Alsahhaf and Att [24]	2016
Kwon et al. [25]	2016

**Table 2:** Data from the literature review in which the parameter of the force used for the extrusion was compared (grey filled box = yes applied, posterior = back elements, anterior = front elements).

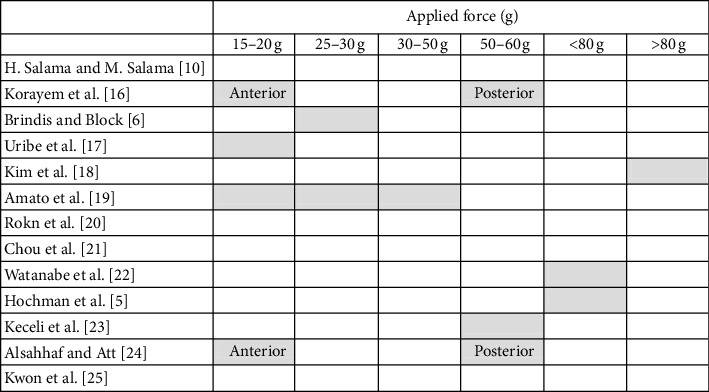

**Table 3:** Data from the literature review in which the parameters of the extrusion speed, the overcorrection (OC), the use of fibrotomy (FB), and the follow-up (FU) were compared (grey filled box = yes applied) (m = months, y = years, X-ray = endoral radiography, CBCT = cone beam computer tomography).

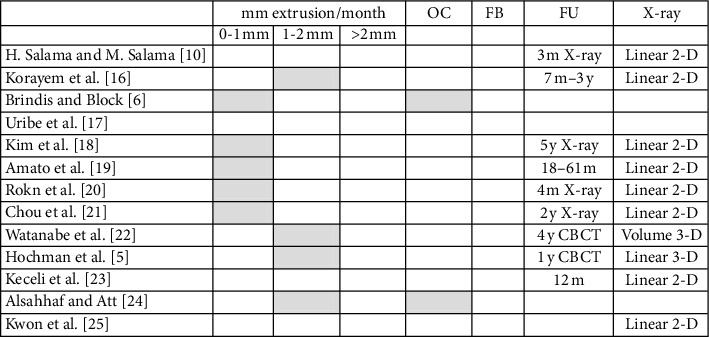

**Table 4:** Data from the literature review in which the parameter of the stabilization times was compared (grey filled box = yes applied).

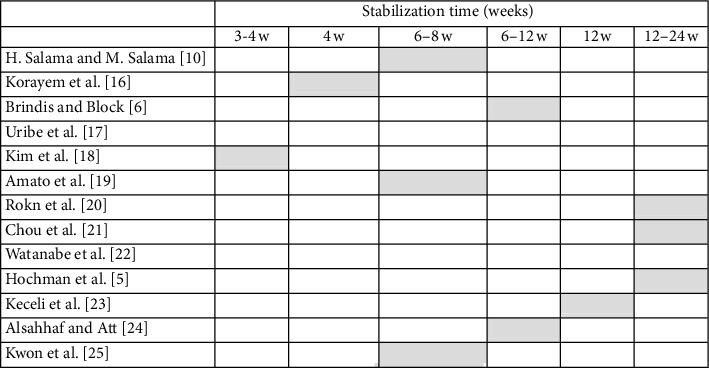

**Table 5:** Results obtained from the measurements of the areas at time T0 and at time T1. All the patients examined showed a significant increase in bone areas with an average value of 31.575 mm^2^ from a minimum of 21.5 mm^2^ to a maximum of 41.7 mm^2^. The teeth problems are reported.

	Tooth problem	Area T0 (mm^2^)	Area T1 (mm^2^)	ΔT0 − T1 (mm^2^)
Subject 1 tooth 4.6	Endo (floor perf)	53.1	30.2	22.9
Subject 2 tooth 4.4	Endo (apical)	39.4	6.9	32.5
Subject 3 tooth 1.4	Perio, PD, BOP, Mb^++^	52.9	11.2	41.7
Subject 4 tooth 4.6	Endo (floor perf)	47.3	5.9	41.4
Subject 5 tooth 1.5	Perio, PD, BOP, Mb^+++^	46.8	14.7	32.1
Subject 6 tooth 1.5	Perio, PD, BOP, Mb^++^	34.2	11.5	22.7
Subject 7 tooth 4.5	Endo (apical)	39.6	18.2	21.4
Subject 8 tooth 2.4	Perio, PD, BOP^++^	45.8	9.5	36.3
Subject 9 tooth 4.5	Perio, PD, BOP, Mb^+++^	49.3	10.2	39.1
Subject 10 tooth 1.1	Endo (int res)	34.6	8.4	26.2
Subject 11 tooth 2.5	Perio, PD, BOP, Mb^++^	41.7	16.9	24.8
Subject 12 tooth 2.1	Endo (int res)	51.5	13.7	37.8
Mean value		44.68333333	13.10833333	31.575
S.D.		6.694208261	6.576052744	7.688376705

**Table 6:** Measurements of distance at time T0 and time T1, of bone augmentation, mean, and standard deviation.

	Distance T0 (mm)	Distance T1 (mm)	ΔT1 − T0 (mm)
Subject 1 tooth 4.6	6.2	10.4	4.2
Subject 2 tooth 4.4	5.4	13.2	7.8
Subject 3 tooth 1.4	4.5	6.6	2.1
Subject 4 tooth 4.6	7.6	11.9	4.3
Subject 5 tooth 1.5	4.6	10.1	5.5
Subject 6 tooth 1.5	7.1	11.9	4.8
Subject 7 tooth 4.5	9.3	13.4	4.1
Subject 8 tooth 2.4	8.5	14.7	6.2
Subject 9 tooth 4.5	4.7	7.2	2.5
Subject 10 tooth 1.1	3.9	9.4	5.5
Subject 11 tooth 2.5	5.2	10.4	5.2
Subject 12 tooth 2.1	4.9	8.3	3.4
Mean value	5.991666667	10.625	4.633333333
SD	1.744319786	2.51183562	1.578453405

## Data Availability

All the data used to support the findings of this study are included within the article.
